# Digital Phenotyping Data to Predict Symptom Improvement and App Personalization: Protocol for a Prospective Study

**DOI:** 10.2196/37954

**Published:** 2022-11-29

**Authors:** Danielle Currey, John Torous

**Affiliations:** 1 School of Medicine Case Western Reserve University Cleveland, OH United States

**Keywords:** digital phenotyping, digital phenotype, mental health, depression, anxiety, smartphone, app, college student, university student, young adult, engagement, digital health, mobile health, mHealth, health app, Technology Acceptance Model, adoption

## Abstract

**Background:**

Smartphone apps that capture surveys and sensors are increasingly being leveraged to collect data on clinical conditions. In mental health, this data could be used to personalize psychiatric support offered by apps so that they are more effective and engaging. Yet today, few mental health apps offer this type of support, often because of challenges associated with accurately predicting users’ actual future mental health.

**Objective:**

In this protocol, we present a study design to explore engagement with mental health apps in college students, using the Technology Acceptance Model as a theoretical framework, and assess the accuracy of predicting mental health changes using digital phenotyping data.

**Methods:**

There are two main goals of this study. First, we present a logistic regression model fit on data from a prior study on college students and prospectively test this model on a new student cohort to assess its accuracy. Second, we will provide users with data-driven activity suggestions every 4 days to determine whether this type of personalization will increase engagement or attitudes toward the app compared to those receiving no personalized recommendations.

**Results:**

The study was completed in the spring of 2022, and the manuscript is currently in review at JMIR Publications.

**Conclusions:**

This is one of the first digital phenotyping algorithms to be prospectively validated. Overall, our results will inform the potential of digital phenotyping data to serve as tailoring data in adaptive interventions and to increase rates of engagement.

**International Registered Report Identifier (IRRID):**

PRR1-10.2196/37954

## Introduction

While COVID-19 restrictions begin to end, the crisis in college mental health continues to expand. Recent large-scale studies suggest that the mental health impact of depression and anxiety for college students continues even in mid-2022 [1]. Digital mental health technologies, especially smartphone apps, are a leading tool to help provide more services to students [2]. Numerous college mental health centers already recommend mental health apps, and many programs are aimed specifically at college students [3]. Despite the clear potential of apps to provide easy-to-access and interactive mental health resources, their impact to date has been limited [4]. One leading barrier has been a lack of engagement; many people quickly abandon apps after only a few days [5]. In this paper, we propose a scalable and data-driven approach to customize daily and weekly app content based on predictive models that enable both personal and automated care.

Smartphone apps are well suited to personalize care as they can gather information related to real-time mental health. Often known as digital phenotyping or smartphone sensing, it is possible, for example, to use signals from a smartphone’s accelerometer to infer sleep behaviors and geolocation to infer mobility patterns. Reviews and research on digital phenotyping in college students suggest that, while digital biomarkers do exist [4], their effect size is likely small. In our prior research [6], we have combined these digital biomarkers with brief smartphone surveys to build predictive models of stress, anxiety, and depression. While we have validated these models retrospectively on different data sets of college students, to date there have been no studies exploring their prospective validity and if customizing an app to offer tailored preventive resources may reduce mental health symptoms. Overall, this work aims to prospectively evaluate a model for participant improvement across the study and compare groups that receive personalized interventions via a digital navigator, automated worker, or neither to explore the Technology Acceptance Model (TAM) in college students.

## Methods

First, we will provide general details about the study, and then, we will address how we plan to achieve these two goals.

### Participants, Technology, and App Use

This study will use the open-source mindLAMP app developed by the Digital Psychiatry lab at Beth Israel Deaconess Medical Center to collect survey and sensor data from college student participants [7]. mindLAMP is an app that facilitates survey, digital phenotyping (see below), and app-based intervention all in one platform that runs on Apple and Android smartphones. In this study, GPS, accelerometer, and screen state data will be collected. In addition, the app will be used to administer surveys and provide cognitive games, mindfulness, and other activities. Like earlier iterations of this study, college students will be recruited via social media to complete a screening survey on REDCap [8]. Given that in-person recruitment remains challenging around COVID-19, online recruitment via social media is practical [9]. To participate, students must be 18 years or older, score 14 or higher on the Perceived Stress Scale (PSS) [10], be enrolled as an undergraduate for the duration of the study, own a smartphone able to run mindLAMP, be able to sign informed consent, and pass the run-in period outlined below. We will not exclude students based on any comorbidities. We aim to recruit at least 100 students to start the study in line with our prior pilot studies and the sample sizes used to generate the model we are testing. Given that the effect size of any personalization efforts remain largely undefined, formal power analysis is more challenging; although, we note that this study is larger than prior digital phenotyping studies for college mental health, which have a mean sample size of 81 [11].

Participants will be sent log-in information for the app and will enter a run-in period. During these 3 days, participants will be asked to complete a survey each day. This run-in period will serve to screen out participants whose devices are not able to capture digital phenotyping data or do not engage with the app at all, and give the study coordinators time to verify that informed consent is signed and dated correctly. The run-in period is designed to help improve overall digital data coverage that is important for validation of the predictive model [12]. After these 3 days, participants who have completed the required surveys and have sufficient GPS data will be moved to the enrollment period of the study. Participants who have not completed the required surveys will be emailed by the study worker automation and given 24 hours to complete these tasks before being automatically discontinued.

### Metrics

Participants will be asked to complete a longer survey each week on the app that includes the Patient Health Questionnaire-9 (PHQ-9) [13], Generalized Anxiety Disorder-7 (GAD-7) [14], PSS [10], UCLA Loneliness Survey [15], Pittsburgh Sleep Quality Index [16], Digital Working Alliance Inventory (DWAI) [17], and TAM-related questions (Table 1) [18].

On the first day of the study, participants will also be asked to complete the Prodromal Questionnaire-16 [19] (Multimedia Appendix 1A). Participants will have a daily survey each morning on the app that asks about sleep duration and sleep quality, and has questions from the PHQ-9, GAD-7, and PSS (Multimedia Appendix 1A). Participants will be compensated for completing the weekly surveys: US $15 for completing one survey between the first and eighth days, US $15 for completing at least one survey between the 8th and 21st days, and finally US $20 for completing at least one more survey between the 21st and 28th days. Students will be paid via Amazon gift card codes.

Throughout the study, engagement will be monitored to ensure that a minimum amount of data is being collected. To promote engagement, the study worker will reach out to participants via email if they have not completed any activities in the past 3 days and encourage them to complete the scheduled activities. If participants have not completed any activities in 5 days, they will be discontinued.

**Table 1:** Questions to explore the TAM. Some questions are part of both the DWAI and the TAM model. All answers are on a Likert scale (0, strongly disagree; 1, disagree; 2, neither agree nor disagree; 3, agree; and 4, strongly agree).

Component of TAM^a^ and questions		From DWAI^b^
**Usefulness**		
	The app supports me to overcome challenges.	Yes
	The app allows me to easily manage my mental health.	No
	The app makes me better informed of my mental health.	No
	The app provides me with valuable information or skills.	No
**Ease**		
	The app is easy to use and operate.	Yes
**Attitude**		
	I trust the app to guide me toward my personal goals.	Yes
	I believe the app tasks will help me to address my problems.	Yes
	The app encourages me to accomplish tasks and make progress.	Yes
	I agree that the tasks within the app are important for my goals.	Yes
**Behavioral intention**		
	I want to use the app daily.	No
	I would want to use it after the study ends.	No

^a^TAM: Technology Acceptance Model.

^b^DWAI: Digital Working Alliance Inventory.

### Activities

**Figure 1:**
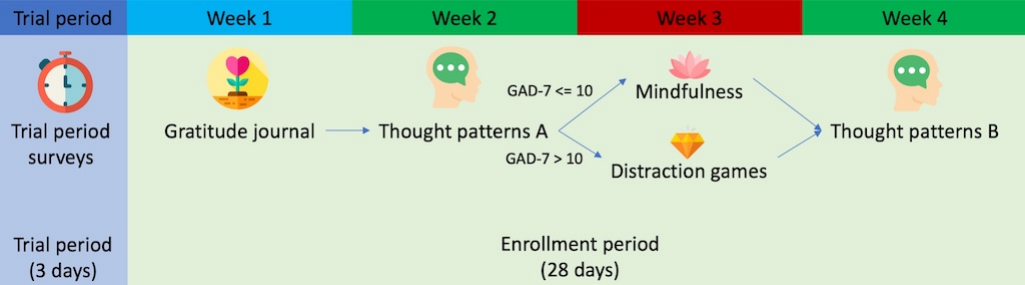
Activities throughout the study. Following a 3-day run-in period, participants will complete different module activities each week. GAD-7: Generalized Anxiety Disorder-7.

All participants will be scheduled for different therapeutic modules each week. The activities are listed in the app under the participant’s daily task feed. The components of the study are shown in Figure 1.

**Figure 2:**
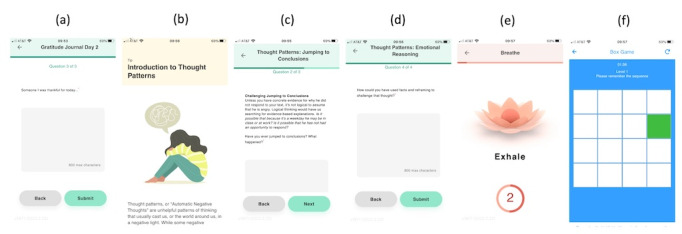
Screenshots of activities in the mindLAMP app including (A) gratitude journal (week 1); (B) the thought patterns learn tip (weeks 2 and 4); (C) a thought patterns activity example (week 2); (D) thought patterns, asking the user to reframe their thought (week 4); (E) a breath activity (week 3); and (F) the spatial span game (week 3).

These modules include content created specifically for college students. For the first week, all participants will be scheduled for gratitude journaling. In the second and fourth weeks, participants will learn about different types of thought patterns and practice recoding and rationalizing their thoughts (Multimedia Appendix 1B). Screenshots of the app modules are shown in Figure 2.

**Figure 3:**
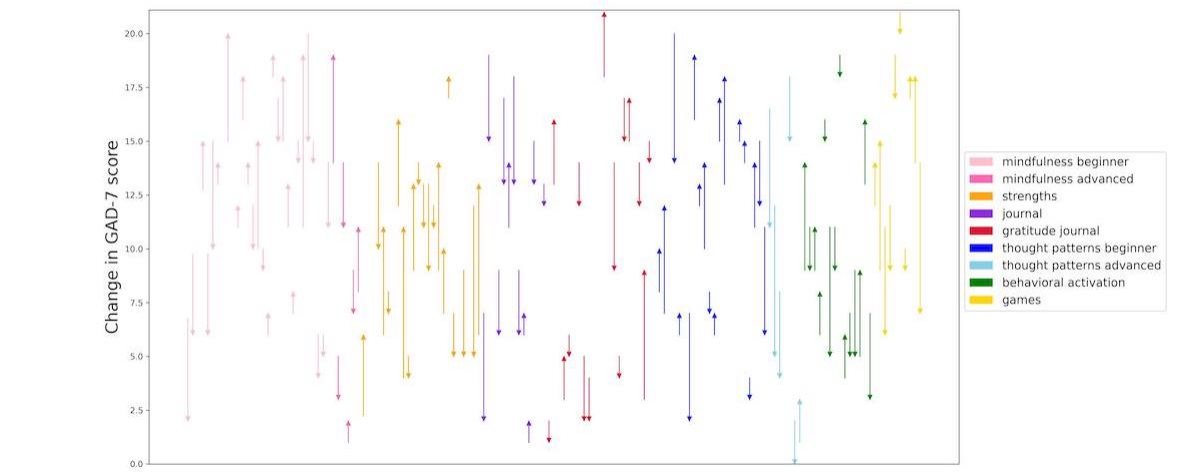
Improvement across different modules from earlier studies. The change in score is shown via the direction of the arrow and the magnitude of the change is shown by the length of the arrow. This highlights the nature of the data used to produce the recommendation model tested in this study. GAD-7: Generalized Anxiety Disorder-7.

We have evaluated improvement (change in GAD-7 scores) in a prior study [20], which is shown in Figure 3. Each participant’s change in GAD-7 is shown by a line going from their start-of-week to end-of-week score. Overall, it is difficult to determine in this small data set if one module is better than the other. However, it seems that participants with higher GAD-7 scores may not improve as much with mindfulness as compared to cognitive distraction games. Thus, in the third week, participants will be scheduled for either mindfulness or cognitive distraction games based on whether they had low (≤10) or high (>10) GAD-7 scores on the initial weekly survey. Participants who do not complete this initial survey within the first week will be discontinued.

### Engagement Theory and Study Design

To address our second aim exploring engagement, we adapted the TAM as a theoretical framework [18]. The TAM is the most widely used model to study engagement around digital health technologies. In the TAM, both perceived ease of use and perceived usefulness influence attitude toward technology, which in turn impacts behavioral intention to use (B) and actual system use. In this study, attitude toward technology will be measured by the DWAI [17]. The predictive models offering tailored resources should increase perceived usefulness and thus attitude toward technology and actual engagement compared to a control group receiving a scheduled set of resources.

**Figure 4:**
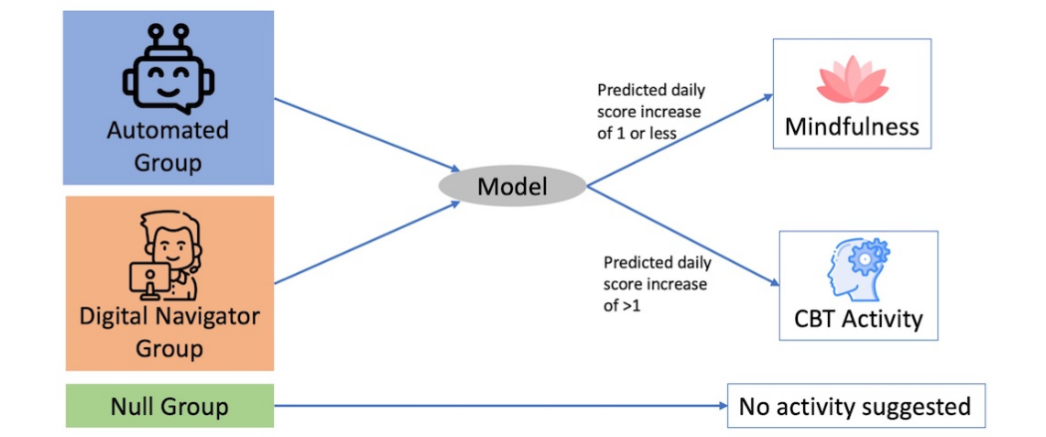
The study will be split into 3 different groups. Activities will be suggested based on model predictions. CBT: cognitive behavioral therapy.

However, increasing perceived usefulness may not be enough, as recent studies suggest the need for a social, or at least human, interaction to drive engagement. It is currently unclear if this interaction would have the largest effect on perceived usefulness, attitude toward technology, or behavioral intention to use, and thus, we will perform an exploratory analysis around this question. The study will be split into three groups. For those in the first group, digital navigators [20] will provide human support and reach out every fourth day to suggest a different module based on whether the algorithm (described below) predicts future symptom worsening or improvement. In this study, navigators are research assistants who have been trained in our 10-hour curriculum on how to provide technical and engagement support for people using health apps [21]. They will use email to communicate with participants, although we automated much of the role for this study as outlined below. For those assigned to the automation arm, modules will be suggested every fourth day by the automated study worker bot via email. Our automation platform will generate emails, and those assigned to the bot group will receive that email, while those assigned to the digital navigator group will have theirs reviewed and signed by such. Finally, for those assigned to the third arm, or the null group, there will be no modules suggested or automation/digital navigator interaction. Study staff will be available to answer any study questions from all participants. The reason for activities being suggested every 4 days is to allow participants to practice the suggested skills and resources, and allow a window for these to impact symptoms. Upon enrollment, participants will be sequentially assigned to one of three groups: the automated group, the digital navigator group, or the null group (Figure 4).

### Machine Learning Model for Engagement Intervention

We present a logistic regression model trained on the passive data features of a prior study of college students to predict whether daily survey scores would increase by one or more (any decrease in mental health). The model will be used to predict every fourth day if there will be an increase in reported symptoms. The model is used to demonstrate the feasibility of applying a data-driven approach to activity suggestions. On these days, students in the digital navigator group and the automated group will receive a suggestion via email for an additional activity to complete from either a digital navigator or the automation worker bot, respectively. On days with an expected increase, a cognitive behavioral therapy–based exercise will be assigned, and on days without an expected increase, a mindfulness exercise will be assigned. These activities will be pulled sequentially from a predefined list and will be different from the weekly activities (Multimedia Appendix 1C). In addition, participants will be asked to complete a 3-question survey about their attitude and behavioral intention toward the app after completing the survey (Multimedia Appendix 1A) as a measure of engagement.

The model was fit using data from the second iteration of the college mental health study using leave-one-patient-out cross-validation on the difference between each of the passive data features from 2 days ago to the previous day to predict a score increase of one or more from the previous to the current day. The implementation of the passive data features used in the model can be found on GitHub [22]. The Scikit-Learn LogisticRegression model was used with a 1:1 ratio of 0.5 [23]. Class weights were balanced, and all input features were standardized. The final model coefficients (Table 2) are an average of the coefficients of each model. The area under the curve (AUC) over all the combined cross-validated folds was 0.648.

**Table 2:** Passive data model coefficients, means, and SDs.

Feature	Coefficient	Mean (SD)
Entropy	–0.07705803	4.132491e-3 (4.193345e-1)
Home time	–0.74001826	–4.256811e5 (2.199408e7)
Screen duration	0.12002379	8.479066e4 (1.127670e7)
GPS data coverage	0.2187653	–2.222512e-3 (2.301561e-1)
Step count	0.11418704	–4.385282e2 (5.877810e3)

### Symptom Improvement Model

To achieve our first aim, we present an additional logistic regression model to predict if participants will improve by at least 25% by the end of the study on the weekly surveys from the average of all features over the course of the study. The model was trained on data from the first iteration of the college study [8] and tested on the second iteration of the college study to test model generalization. The AUC scores are shown in Table 3. The features used in the model and a table of nonzero model coefficients can be found in Multimedia Appendix 1D.

Both previous versions of the study recruited college students to participate in a 28-day study taking daily and weekly surveys. Differences included the time the study was performed (version 1 collected data from December 2020 to May 2021, and version 2 collected data from November to December 2021) and the module activities (version 1 had no assigned activities, and version 2 had four set modules: thought patterns, journaling, mindfulness, and cognitive distraction games).

**Table 3:** Model performance for the improvement model. Results are shown for the second college data set from a model trained on the first college data set.

Survey	Area under the receiver operating characteristic curve
Patient Health Questionnaire-9	0.647
Generalized Anxiety Disorder-7	0.738
Perceived Stress Scale	0.640
UCLA Loneliness Scale	0.835
Pittsburgh Sleep Quality Index	0.634

### End of the Study

The activity schedule will finish after 28 days in the enrollment period. However, if participants have not completed their final weekly survey, they will be given up to 4 additional days to complete this survey and receive compensation. At 32 days, all remaining participants will be marked as completed, and their sensor data collection will be turned off.

### Study Automation and Data Coverage

**Figure 5:**
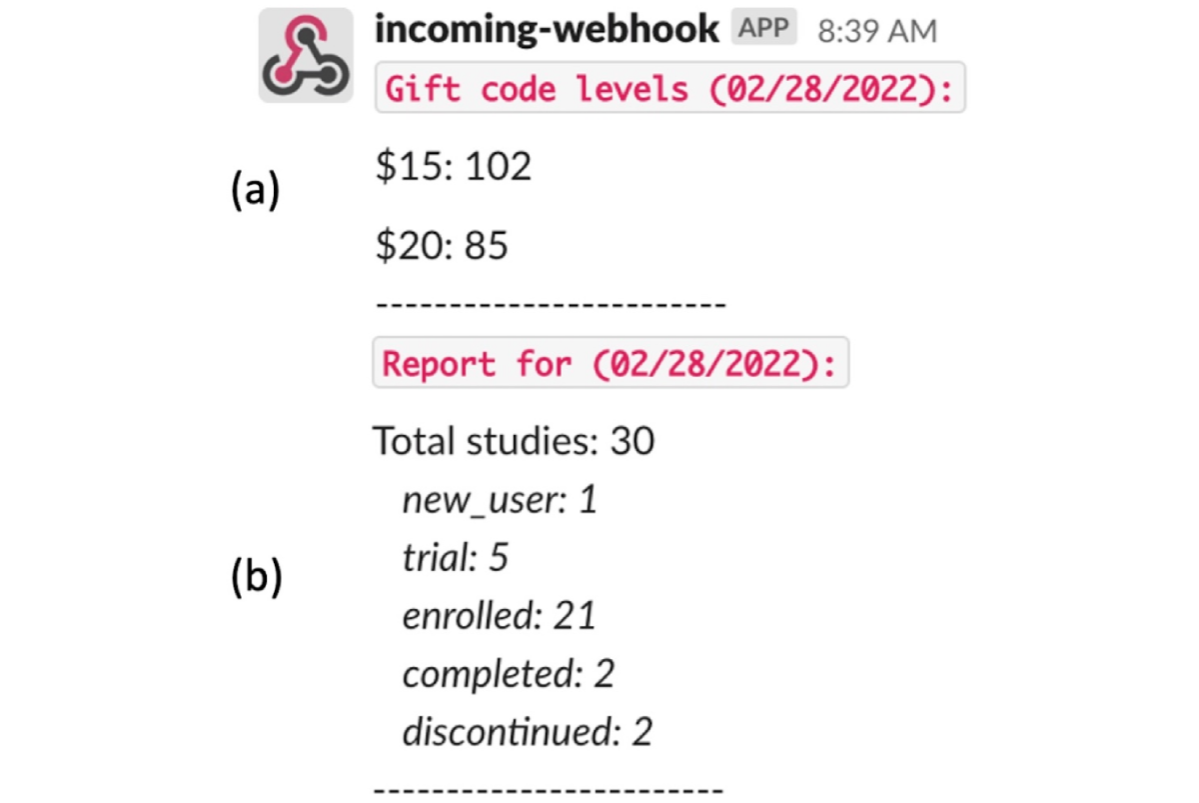
Slack notifications from the study worker: (A) lists levels of available gift codes for payment and (B) reports the number of participants in each phase of the study.

To enable scalable research, we will build upon the digital study infrastructure used in our prior studies [12]. All parts of the study will be automated via workers implemented in Python. We have added new features to the codebase, including a worker that will update a Google Sheet with study information such as the status of different participants in the study, payment form completion, and which activities have been assigned. In addition, automated Slack notifications will be sent to the team to help manage the study (Figure 5). These improvements will provide an easy way for the study team to track study progress.

**Figure 6:**
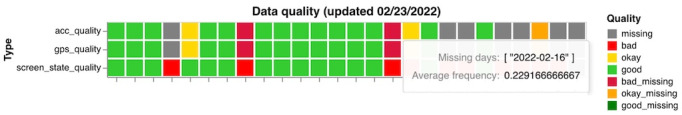
Passive data coverage graphs. Rows show accelerometer coverage (acc_quality) GPS coverage (gps_quality) and screen state coverage (screen_state_quality). Further details can be found on Github [22].

Passive and active data coverage will additionally be monitored throughout the study via Slack notifications sent to the study team and graphs on the data portal (Figure 6). Graphs will include participant GPS, accelerometer, and screen state coverage over the past week, days since the last activity, previous week’s module completion, and previous week’s daily/weekly survey counts. These graphs will allow researchers to monitor for any study-wide data collection issues and track overall participant engagement at a high level.

In addition to these researcher-facing metrics, participants will receive a weekly progress email telling them their streak, number of weekly and daily surveys completed, and module completion to promote engagement. The code for the study workers can be found on GitHub [24].

### Safety

For any participants who indicate thoughts related to self-harm or suicide as noted by a score of 3 on question number nine of the PHQ-9, an alert will be sent to study staff by the automated study worker, and the principal investigator or covering licensed clinicians will reach out to the student within the same business day to conduct a safety assessment. If the student cannot be reached via phone or email after 24 hours, we will notify the local student mental health services. At the same time a participant records an elevated thought of self-harm or suicide, the app also displays a reminder that it is not a replacement for emergency care and that study staff cannot respond in real time, and provides links and phone numbers to resources.

### Ethics Approval

This study was approved by the Beth Israel Deaconess Medical Center institutional review board (protocol 2020P000310). Data is not available to share, but the smartphone app and feature processing code are.

## Results

The first key goal of this work is to prospectively evaluate a model predicting improvement across the study. Second, we aim to analyze the effectiveness of suggesting personalized modules to participants. We will compare the improvement of the automated and digital navigator groups to see if there is a significant effect of having a person versus artificial intelligence delivering information. We will also compare the automated and digital navigator groups with the null group to see whether suggested modules and interaction during the study increases engagement or improvement. As a secondary outcome, we will perform an ANOVA analysis to compare the TAM questions across the three study groups, acknowledging that this type of analysis is novel and that the results of individual questions will be challenging to compare to prior literature. The study was completed in the spring of 2022, and the results will be published with JMIR Publications.

## Discussion

The results of this study will inform both data science and clinical engagement questions around digital college mental health. First, by prospectively testing our algorithms on a unique sample, we can determine both their reliability and validity. Second, by assessing engagement outcomes with digital navigators versus automations versus a control group, we can learn how to best increase the use of apps and build mechanistic understanding using the TAM.

While many smartphone digital phenotyping biomarkers and algorithms have been proposed across the mental health field and even specifically for college mental health [11], none have ever been prospectively validated. In our prior research, we have been able to retrospectively validate findings, used to inform this study’s methods, on older data sets [6,8].

Beyond their predictive ability, the results around the validity of the digital phenotyping biomarkers hold potential for advancing adaptive interventions [25]. A key component of adaptive interventions is the tailoring variable that is used to customize treatment at each decision time point [26]. While most tailoring variables are static (eg mood score above a predetermined threshold at a certain time), digital phenotyping biomarkers could serve as more dynamic tailoring variables that would enable more personalized treatments. Given that smartphones themselves can serve as platforms to offer these adaptive interventions, the results around optimal tailoring variables are highly relevant.

The digital navigator group, as well as the control group, offer useful comparisons that must be considered. Digital navigators are increasingly used to increase engagement although at the price of greater scalability. Still, most apps today are not supported by either digital navigators or algorithms, so comparing outcomes to a control group can help assess any potential benefit. Additionally, it remains difficult to determine which activities are best for participants or which interventions should be assigned in real time in response to passive data changes. This challenge makes it difficult to truly personalize app recommendations. However, it may be the case that providing expert or data-driven suggestions to the participant introduces a placebo effect that improves engagement and attitude toward the app regardless of the actual usefulness of the activity. Although difficult to explore in this study, comparing different app activities is an interesting area of future work.

Further secondary outcomes related to the TAM can also help inform mechanistic-based understanding of engagement. While many prior studies, including our own, have examined outcomes like usability, fewer have explored why apps are engaging. Even if our results are negative around engagement, learning how TAM scores change over time and correlate to rates of app use will inform how future versions of mindLAMP can be improved.

There are limitations to this protocol. For secondary outcomes regarding automated interventions, given that our model here has a low AUC, the results will have to be interpreted with caution. While our study is designed to prospectively validate the symptom algorithm, it is not powered around the secondary engagement outcomes. This is in part due to the effect size for different engagement strategies like digital navigators and personalization remaining poorly defined. Thus, our results can help inform future study design.

Like our prior studies, our research is fully reproducible. We offer details of our recruitment process and procedures in this paper that outlines details of our recruitment, screening, and data coverage procedures [12]. The mindLAMP app remains open-source software currently deployed at over 50 clinical sites worldwide, and our algorithms are also publicly accessible via GitHub [27]. This enables others to validate and expand upon our work transparently. While not a study outcome, the decentralized clinical trial mechanism used in this study offers a practical example of how digital phenotyping research can be done in a remote yet scalable manner.

## Appendix

Multimedia Appendix 1 Additional figures and supplementary material.

## Abbreviations

AUC: area under the curve
DWAI: Digital Working Alliance Inventory
GAD-7: Generalized Anxiety Disorder-7
PHQ-9: Patient Health Questionnaire-9
PSS: Perceived Stress Scale
TAM: Technology Acceptance Model

## Data Availability

Data from this study is not available given the personal identifiable nature of the information. However, the mindLAMP app and processing code are freely available.
