# Supplementary material for: Digital Phenotyping Data to Predict Symptom Improvement and App Personalization: Protocol for a Prospective Study
Source: JMIR Res Protoc. 2022 Nov 29;11(11):e37954. doi: 10.2196/37954 (PMC9748794; doi:10.2196/37954)
Supplement: Multimedia Appendix 1 [file resprot_v11i11e37954_app1.docx]

**Supplementary material**

# Appendix A:

*A.1*

Daily survey questions. Answers for PHQ-9, GAD-7, and PSS are on a Likert scale (0 – Not at all, 1 – Several times, 2 – More than half the time, 3 - Nearly all the time). Sleep quality is on a scale of 0 (best) to 9 (worst).

| **Survey** | **Question** |
| --- | --- |
|  | Total score: sum of PHQ-9, GAD-7, and PSS questions |
| PHQ-9 | I feel down today. |
|  | Today I felt little interest or pleasure. |
| GAD-7 | Today I had trouble relaxing. |
|  | I have been worrying too much today. |
| PSS | I feel like I cannot cope with all of the things I have to do today. |
| Sleep | What time did you fall asleep last night? |
|  | What time did you wake up this morning? |
|  | Overall, how would you rate your sleep last night? |

*A.2*

Weekly survey questions. Answers are on a Likert scale (0 – Not at all, 1 – Several days, 2 – More than half the days, 3 - Nearly every day) except for some PSQI questions (which are on a 0 to 3 scale where 3 is the worst and answers relate to the specific question) and D-WAI and TAM which are also on a Likert scale (0 – Strongly disagree, 1 – Disagree, 2 – Neither agree nor disagree, 3 – Agree, 4 – Strongly agree).

| **Survey** | **Question** |
| --- | --- |
| PHQ-9 | Over the past week, I have had little interest or pleasure in doing things. |
|  | Over the past week, I have felt down, depressed, or hopeless. |
|  | Over the past week, I have had trouble falling asleep, starting asleep, or sleeping too much. |
|  | Over the past week, I have felt tired or have had little energy. |
|  | Over the past week, I have experienced poor appetite or overeating |
|  | Over the past week, I have felt bad about myself, or that I am a failure or have let down myself or my family. |
|  | Over the past week, I have had trouble concentrating on things such as reading the newspaper or watching television. |
|  | Over the past week, I have found myself moving or speaking so slowly that other people could have noticed. Or the opposite - being so fidgety or restless that I have been moving around a lot more than usual. |
|  | Over the past week, I have had thoughts that I would be better off dead, or thoughts of hurting myself. |
| GAD-7 | Over the past week, I have felt nervous, anxious, or on edge. |
|  | Over the past week, I have not been able to stop or control worrying. |
|  | Over the past week, I have been worrying too much about different things. |
|  | Over the past week, I have had trouble relaxing. |
|  | Over the past week, I have felt so restless that it's hard to sit still. |
|  | Over the past week, I have felt myself becoming easily annoyed or irritable. |
|  | Over the past week, I have felt afraid as if something awful might happen. |
| PSS | In the last week, how often have you been upset because of something that happened unexpectedly? |
|  | In the last week, how often have you felt that you were unable to control the important things in your life? |
|  | In the last week, how often have you felt nervous and stressed? |
|  | In the last week, how often have you felt confident about your ability to handle your personal problems? |
|  | In the last week, how often have you felt that things were going your way? |
|  | In the last week, how often have you found that you could not cope with all the things that you had to do? |
|  | In the last week, how often have you been able to control irritations in your life? |
|  | In the last week, how often have you felt that you were on top of things? |
|  | In the last week, how often have you been angered because of things that happened that were outside of your control? |
|  | In the last week, how often have you felt difficulties were piling up so high that you could not overcome them? |
| UCLA Loneliness | I am unhappy doing so many things alone. |
|  | I have nobody to talk to. |
|  | I cannot tolerate being so alone. |
|  | I lack companionship. |
|  | I feel as if nobody really understands me. |
|  | I find myself waiting for people to call or write. |
|  | There is no one I can turn to. |
|  | I am no longer close to anyone. |
|  | My interests and ideas are not shared by those around me. |
|  | I feel left out. |
|  | I feel completely alone. |
|  | I am unable to reach out and communicate with those around me. |
|  | My social relationships are superficial. |
|  | I feel starved for company. |
|  | No one really knows me well. |
|  | I feel isolated from others. |
|  | I am unhappy being so withdrawn. |
|  | It is difficult for me to make friends. |
|  | I feel shut out and excluded by others. |
|  | People are around me but not with me. |
| PQ-16 | I feel uninterested in the things I used to enjoy. |
|  | I often seem to live through events exactly as they happened before (déjà vu). |
|  | I sometimes smell or taste things that other people can’t smell or taste. |
|  | I often hear unusual sounds like banging, clicking, hissing, clapping or ringing in my ears. |
|  | I have been confused at times whether something I experienced was real or imaginary. |
|  | When I look at a person, or look at myself in a mirror, I have seen the face change right before my eyes. |
|  | I get extremely anxious when meeting people for the first time. |
|  | I have seen things that other people apparently can't see. |
|  | My thoughts are sometimes so strong that I can almost hear them. |
|  | I sometimes see special meanings in advertisements, shop windows, or in the way things are arranged around me. |
|  | Sometimes I have felt that I’m not in control of my own ideas or thoughts. |
|  | Sometimes I feel suddenly distracted by distant sounds that I am not normally aware of. |
|  | I have heard things other people can't hear like voices of people whispering or talking. |
|  | I often feel that others have it in for me. |
|  | I have had the sense that some person or force is around me, even though I could not see anyone. |
|  | I feel that parts of my body have changed in some way, or that parts of my body are working differently than before. |
| PSQI | How often is it that you cannot get to sleep within 30 minutes? |
|  | How often is it that you wake up in the middle of the night or early morning? |
|  | How often have you had trouble sleeping because you cannot breathe comfortably? |
|  | How often have you had trouble sleeping because you cough or snore loudly? |
|  | How often have you had trouble sleeping because you feel too hot? |
|  | How often have you had trouble sleeping because you have bad dreams? |
|  | How often have you had trouble sleeping because you have pain? |
|  | During the past week, how would you rate your sleep quality overall? |
|  | During the past week, how often have you taken medicine to help you sleep (prescribed or "over the counter")? |
|  | During the past week, how often have you had trouble staying awake while driving, eating meals, or engaging in social activity? |
|  | During the past week, how much of a problem has it been for you to keep up enthusiasm to get things done? |
| DWAI | I trust the app to guide me towards my personal goals. |
|  | I believe the app tasks will help me to address my problems. |
|  | The app encourages me to accomplish tasks and make progress. |
|  | I agree that the tasks within the app are important for my goals. |
|  | The app is easy to use and operate. |
|  | The app supports me to overcome challenges. |
| TAM (not in DWAI) | The app allows me to easily manage my mental health. |
|  | The app makes me better informed of my mental health. |
|  | The app provides me with valuable information and/or skills. |
|  | I want to use the app daily. |
|  | I would want to use it after the study ends. |

*A.3*

Check-in survey questions. The first question is a Boolean response (yes / no). The second and third questions are on a Likert scale (0 – Strongly disagree, 1 – Disagree, 2 – Neither agree nor disagree, 3 – Agree, 4 – Strongly agree).

| **TAM component** | **Question** |
| --- | --- |
|  | Did you complete the suggested activity? |
| A | I agree that the tasks within the app are important for my goals. |
| B | I would want to complete this activity again. |

**Appendix B:**

*B.1*

Module activities by week.

| **Day** | **Week 1:**  **Gratitude Journal** | **Week 2:**  **Thought Patterns A** | **Week 3:**  **Mindfulness** | **Week 3:**  **Cognitive Games** | **Week 4:**  **Thought Patterns B** |
| --- | --- | --- | --- | --- | --- |
| 1 | Learn tip + Gratitude Day 1 | Learn tip | Mindfulness Day 1 + Learn tip | Jewels | Learn tip +  Fortune Telling |
| 2 | Gratitude Day 2 | Catastrophizing | Morning Mindfulness + Mindfulness Day 2 | Spatial Span | Minimizing |
| 3 | Gratitude Day 3 | All or Nothing | Morning Mindfulness + Mindfulness Day 3 | Jewels | Emotional Reasoning |
| 4 | Gratitude Day 4 | Record, Rationalize, Replace | Morning Mindfulness + Mindfulness Day 4 | Spatial Span | Record, Rationalize, Replace |
| 5 | Gratitude Day 5 | Jumping to Conclusions | Morning Mindfulness + Mindfulness Day 5 | Jewels | Should-y Thinking |
| 6 | Gratitude Day 6 | Mind Reading | Morning Mindfulness + Mindfulness Day 6 | Spatial Span | Personalization |
| 7 |  | Record, Rationalize, Replace | Morning Mindfulness | Jewels | Record, Rationalize, Replace |

**Appendix C:**

*C.1*

Digital navigator activities to suggest to participants every 4 days. These will be presented in order based on module predictions.

| **Number** | **No score increase** | **Score increase** |
| --- | --- | --- |
| 1 | Anchoring Ambiance | Socratic Questions |
| 2 | Calming your Body | Challenging Self-Criticism |
| 3 | Forest and Nature Sounds | Focus on the Positive |
| 4 | Inner Teacher | Strengths |
| 5 | Loving Kindness | Behavioral Activation |
| 6 | Mountain Meditation | Behavioral Experiment |

*C.2*

Email Scripts.

*Every 4 days (Digital Navigator):*

Hello,

Nice job in the past couple of days! Based on your data, we’d like to suggest another activity for you. ________ can be found in the ________ tab. If you complete this activity, please make sure to complete the Check-in survey to let us know what you thought. Let me know if you have any questions or concerns!

Best,

X

*Every 4 days (Study bot):*

Hello,

Nice job in the past couple of days! Based on your data, we’d like to suggest another activity for you. ________ can be found in the ________ tab. If you complete this activity, please make sure to complete the Check-in survey to let us know what you thought. Feel free to email [collegestudy@bidmc.harvard.edu](mailto:collegestudy@bidmc.harvard.edu) if you have any questions!

Best,

Marvin (A Friendly College Study Bot)

*No active data in the past 3 days (Digital Navigator):*

Hello,

We noticed that you haven’t been very active in mindLAMP as of late. Make sure to complete those Daily and Weekly surveys and the module activities that show on your feed each day. Unfortunately, if we don’t see increased participation, we’ll need to discontinue you from the study. Please let us know if you have any questions!

Best,

X

**Appendix D:**

*D.1*

Improvement model coefficients. Only non-zero coefficients are listed.

|  | **PHQ-9** | **GAD-7** | **PSS** | **UCLA** | **PSQI** |
| --- | --- | --- | --- | --- | --- |
| Over the past week, I have felt down, depressed, or hopeless. | -0.133657 | -0.007115 | -0.063245 | -0.053104 |  |
| Over the past week, I have had trouble falling asleep, staying asleep, or sleeping too much. | -0.040673 |  |  |  |  |
| Over the past week, I have felt tired or have had little energy. |  |  |  |  | -0.144456 |
| Over the past week, I have experienced a poor appetite or overeating. |  | 0.089340 | -0.043387 |  |  |
| Over the past week, I have felt bad about myself or that I am a failure or have let down myself or my family. |  |  | 0.180907 |  |  |
| Over the past week, I have had trouble concentrating on things such as reading the newspaper or watching television. |  |  | 0.324889 |  |  |
| Over the past week, I have found myself moving or speaking so slowly that other people could have noticed. Or the opposite - being so fidgety or restless that I have been moving around a lot more than usual. |  |  | -0.075721 |  | -0.011927 |
| Over the past week, I have had thoughts that I would be better off dead, or thoughts of hurting myself. | 0.067468 |  | 0.647949 |  |  |
| Over the past week, I have had trouble relaxing. |  |  | 0.604253 |  |  |
| Over the past week, I have felt so restless that it is hard to sit still. |  |  | -0.397300 |  |  |
| Over the past week, I have felt myself becoming easily annoyed or irritable. |  | -0.000829 | -0.022915 |  |  |
| Over the past week, I have felt afraid as if something awful might happen. |  |  | -0.249266 |  |  |
| PSS | -0.073616 | -0.068288 |  |  |  |
| Over the past week, how often have you been upset because of something that happened unexpectedly. |  |  | -0.167293 |  |  |
| In the last week, how often have you felt confident about your ability to handle your personal problems? |  | -0.046093 | -0.420182 |  | -0.333342 |
| In the last week, how often have you been able to control irritations in your life? |  |  | -0.098208 |  | -0.024570 |
| In the last week, how often have you been angered because of things that happened that were outside of your control? |  |  |  |  | 0.112193 |
| In the last week, how often have you felt that things were going your way? | -0.026022 |  | -0.518583 |  |  |
| In the last week, how often have you felt that you were unable to control the important things in your life? | -0.025609 |  |  |  |  |
| In the last week, how often have you felt nervous and stressed? |  | 0.014835 |  |  |  |
| In the last week, how often have you felt that you could not cope with all the things you had to do? | -0.041151 |  | -0.247050 |  |  |
| In the last week, how often have you felt that difficulties were piling up so high that you could not overcome them? | -0.081862 |  |  |  |  |
| UCLA |  |  |  | -0.176555 |  |
| I have nobody to talk to. |  |  |  |  | 0.002971 |
| I cannot tolerate being so alone. |  |  | -0.583714 |  |  |
| I lack companionship. |  |  | -0.039447 |  | 0.154583 |
| I feel as if no one really understands me. |  |  | -0.298134 | -0.024247 |  |
| There is no one I can turn to. |  |  | 0.015274 |  |  |
| I am no longer close to anyone. |  |  |  | -0.086054 |  |
| My interests and ideas are not shared by those around me. | -0.032290 |  | -0.223867 | -0.145194 |  |
| I feel left out. |  |  |  | -0.171426 |  |
| I feel starved for company. |  |  |  | -0.183726 |  |
| No one really knows me well. |  |  |  | -0.058888 |  |
| I feel isolated from others. |  |  |  | -0.006047 |  |
| I feel shut out and excluded by others. |  |  |  | -0.017604 |  |
| I find myself waiting for people to call or write. |  |  |  |  | 0.503658 |
| I am unable to reach out and communicate with those around me. |  |  |  |  | 0.155351 |
| I feel isolated from others. |  |  | -0.145731 |  |  |
| My social relationships are superficial. |  |  |  |  | 0.044600 |
| It is difficult for me to make friends. |  |  |  |  | -0.031167 |
| I often seem to live through events exactly as they happened before. |  |  | 0.010295 |  |  |
| I often smell or taste things that other people can’t smell or taste. |  |  | 0.476440 |  |  |
| I have been confused at times whether something I experienced was real or imaginary. |  |  | -0.128605 |  | -0.436523 |
| I have seen things that other people apparently can’t see. |  |  | -0.019311 |  |  |
| I often hear unusual sounds like banging, clicking, hissing, clapping, or ringing in my ears. |  |  |  |  | -0.145346 |
| When I look at a person, or look at myself in a mirror, I have seen the face change right before my eyes. |  |  |  |  | 0.006436 |
| Sometimes I see special meanings in advertisements, shop windows, or in the way things are arranged around me. |  |  | -0.337531 |  | 0.519253 |
| Sometimes I have felt that I am not in control of my own ideas or thoughts. |  |  | -0.110485 |  | -0.653799 |
| I have heard things other people can't hear like voices of people whispering or talking. |  |  |  |  | -0.027569 |
| I often feel that others have it in for me. |  |  | 0.421697 |  | -0.795583 |
| I have had the sense that some person or force is around me, even though I could not see anyone. |  |  |  |  | -0.177277 |
| I feel that parts of my body have changed in some way, or that parts of my body are working differently than before. |  |  | 0.227194 |  | -0.122222 |
| My thoughts are sometimes so strong that I can almost hear them. |  | -0.058933 |  |  |  |
| How often is it that you cannot get to sleep in 30 minutes? |  |  | 0.516843 |  | -0.218500 |
| How often is it that you wake up in the middle of the night or the early morning? |  |  | -0.056563 |  |  |
| How often have you had trouble sleeping because you cannot breathe comfortably? |  |  |  |  | 0.336447 |
| How often have you had trouble sleeping because you feel too hot? |  |  |  |  | -0.371604 |
| How often is it that you have trouble sleeping because of bad dreams? |  |  | 0.203694 |  | 0.219557 |
| During the past week, how often have you had trouble staying awake while driving, eating meals, or engaging in social activity? |  |  |  |  | -0.214490 |
| During the past week, how much of a problem has it been for you to keep up enthusiasm to get things done? | -0.091458 |  |  |  |  |
| PSQI Component Score | -0.202680 | -0.132248 |  |  | -0.191076 |
| PSQI component 2 |  |  |  |  | 0.0967378 |
| PSQI component 3 |  |  | 0.096345 |  |  |
| PSQI component 4 |  |  | 0.222239 |  | 0.287547 |
| PSQI component 5 |  |  | 0.115443 |  |  |
| PSQI component 7 |  |  | 0.016907 |  |  |
| I trust the app to guide me toward my personal goals. |  |  | 0.070091 |  |  |
| I believe that the tasks in the app will help me address my problems. |  |  | -0.071298 |  |  |
| The app encourages me to accomplish tasks and make progress. |  |  | -0.028171 |  | 0.173656 |
| I agree that the tasks within the app are important for my goals. |  |  |  |  | 0.019432 |
| The app is easy to use and operate. |  |  | -0.054996 |  |  |
| Today I feel little interest or pleasure. |  | -0.132751 | -0.400589 |  |  |
| Number of activities |  |  | -0.474707 |  | -0.332787 |
| Home time |  |  | -0.474564 |  |  |
| Screen duration |  |  | -0.174129 |  | -0.185095 |
| GPS data quality |  |  | 0.151978 |  | 0.108379 |
